# Polymyxin E Induces Rapid *Paenibacillus polymyxa* Death by Damaging Cell Membrane while Ca^2+^ Can Protect Cells from Damage

**DOI:** 10.1371/journal.pone.0135198

**Published:** 2015-08-07

**Authors:** Zhiliang Yu, Yuanning Cai, Wangrong Qin, Jianxun Lin, Juanping Qiu

**Affiliations:** 1 College of Biological and Environmental Engineering, Zhejiang University of Technology, Hangzhou, China; 2 Department of Electrical Engineering, Columbia University, New York, United States of America; Indian Institute of Science, INDIA

## Abstract

Polymyxin E, produced by *Paenibacillus polymyxa*, is an important antibiotic normally against Gram-negative pathogens. In this study, we found that polymyxin E can kill its producer *P*. *polymyxa*, a Gram-positive bacterium, by disrupting its cell membrane. Membrane damage was clearly revealed by detecting the leakage of intracellular molecules. The observation using scanning electron microscopy also supported that polymyxin E can destroy the cell membrane and cause an extensive cell surface alteration. On the other hand, divalent cations can give protection against polymyxin E. Compared with Mg^2+^, Ca^2+^ can more effectively alleviate polymyxin E-induced damage to the cell membrane, thus remarkably increasing the *P*. *polymyxa* survival. Our findings would shed light on a not yet described bactericidal mechanism of polymyxin E against Gram-positive bacteria and more importantly the nature of limited fermentation output of polymyxin E from *P*. *polymyxa*.

## Introduction

Polymyxin E (colistin), a nonribosomal peptide with molecular weight of approximately 1200 Da, is biosynthesized by *Paenibacillus polymyxa*, a Gram-positive bacterium [1–3]. Since 1959, colistin has been used for treatment of the infection caused by Gram-negative pathogens [4, 5]. However, its clinical use was soon limited due to its serious nephrotoxicity and neurotoxicity, leading to almost complete replacement by less-toxic aminoglycosides and antipseudomonal antibiotics. Recently, the interest in its clinical application has been revived due to the emergence of multidrug-resistant Gram-negative pathogens, in which some strains are resistant to almost all currently available antibiotics, leaving very limited choices for antimicrobial therapy. In many such cases, colistin is considered as one of the last-line available options against Gram-negative “superbugs” [6–8].

The basic structure of colistin is a cyclic heptapeptide with a tripeptide side chain acylated by a fatty acid at its amino terminus [9, 10]. The positively charged colistin is shown to have a narrow antibacterial spectrum, mainly against the Gram-negatives, while almost no effect on the Gram-positives, fungi and anaerobic bacteria is found [11]. This is probably due to its selective binding with negatively charged lipid A, the hydrophobic anchor of lipopolysaccharide (LPS) on the outer membrane (OM) of Gram-negative bacteria. Upon initial electrostatic interaction, the hydrophobic *N*-terminal fatty acyl chain and the D-Leu^6^-L-Leu^7^ segment of the colistin molecule will insert into OM. This insertion will weaken the packing of adjacent lipid A and induce the expansion of OM monolayer [12, 13]. Subsequently, it will penetrate OM via a ‘self-promoted uptake’ mechanism [14, 15] and disrupt the physical integrity of the phospholipid bilayer on the inner membrane (IM) [2], eventually causing the IM lysis and cell death.

Compared to extensive studies on colistin’s antibacterial activity against Gram-negative bacteria, little is known about its bactericidal activity against Gram-positive ones. Yet, in some cases, there are studies which report that colistin can kill Gram-positive bacteria [16–20], such as *Staphylococcus aureus*, *Streptococcus agalactiae*, *Bacillus cereus*, *Bacillus subtilis* and *Listeria monocytogenes*. However, the bactericidal mechanism of colistin against Gram-positive bacteria is not very clear yet. In this study, we found that colistin can also kill its producer *P*. *polymyxa*, a Gram-positive bacterium, through disruption of its cell membrane. To our knowledge, it is the first report showing colistin can cause cell death of *P*. *Polymyxa* via inducing leakage of the intracellular molecules. We also investigated the effect of divalent cations on protection of the cell membrane. It was found that Ca^2+^ is more effective than Mg^2+^ in alleviating the colistin-induced damage. Since colistin is biosynthesized by *P*. *polymyxa*, its bactericidal activity to its producer would potentially repress its accumulation in *P*. *polymyxa* during fermentation. Therefore, our findings not only enrich our understanding of colistin’s bactericidal activity to Gram-positive bacteria, but also help to improve its fermentation output in the future.

## Results

### Bactericidal activity of colistin against *P*. *polymyxa*

**Fig 1:**
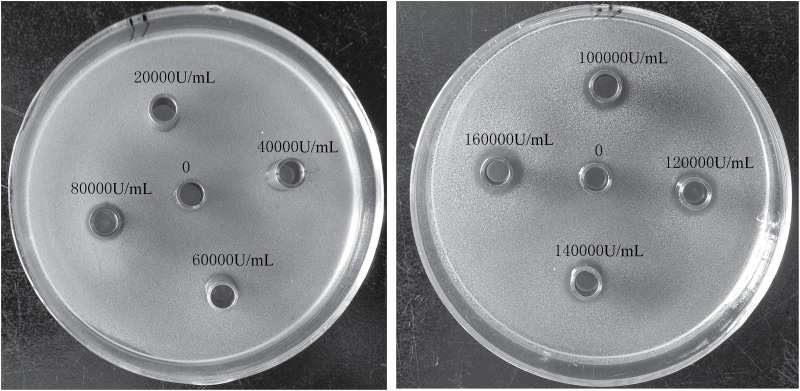
Growth-inhibition zones of *P*. *polymyxa* caused by polymyxin E at various concentrations from 0 to 1.6×10^5^ U/mL.

Bactericidal activity of colistin against *P*. *polymyxa* was tested at various concentrations using disk diffusion assay (Fig 1). Results show that colistin at concentration <6×10^4^ U/mL gives no clear inhibition zone, whereas colistin at concentration >8×10^4^ U/mL displays bactericidal activity against *P*. *polymyxa* as indicated by visible growth-inhibition zones. As the colistin concentrations increase from 8×10^4^ U/mL to 1.6×10^5^ U/mL, the diameter of the inhibition zones is positively correlated with the colistin concentration. In all the following experiments, 1.6×10^5^ U/mL colistin was used to treat *P*. *polymyxa*, unless otherwise indicated.

### Protection of *P*. *polymyxa* against growth-inhibition by divalent cations

**Fig 2:**
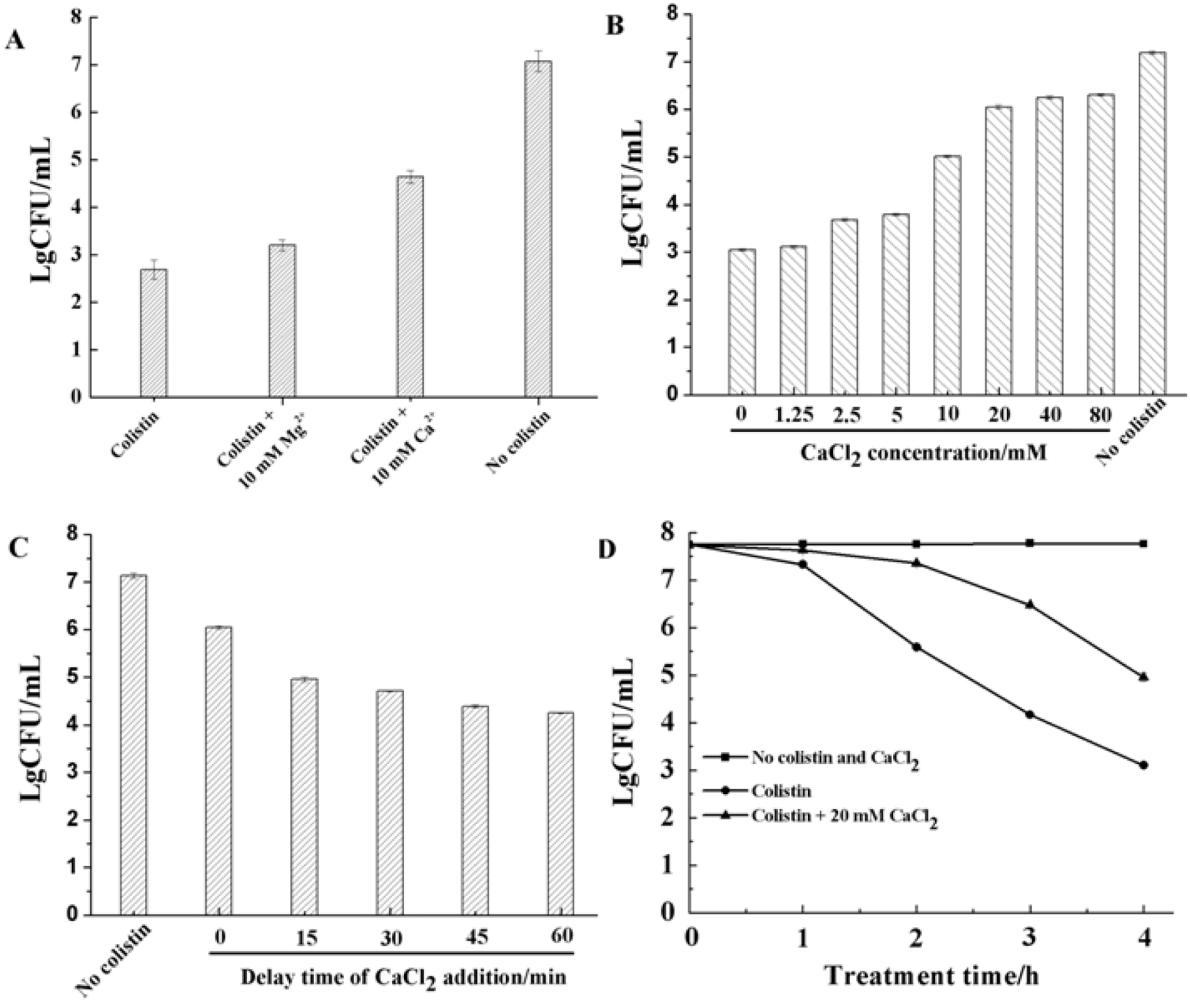
Protection of *P*. *polymyxa* against growth-inhibition by divalent cations at 1.6×10^5^ U/mL colistin. (A) protection difference between Ca^2+^ and Mg^2+^; (B) dependence of Ca^2+^ concentration on protection. (C) effect of delay time of Ca^2+^ addition on protection. (D) time-dependent protection of Ca^2+^ on *P*. *polymyxa* from colistin.

To investigate the potential protective effect of divalent cations on the survival of *P*. *polymyxa* when treated with colistin, CaCl_2_ or MgCl_2_ was added to the treatment. Fig 2A shows that colistin causes the significant decrease of LgCFU/mL from 7.07 to 2.69, while both Ca^2+^ and Mg^2+^ can relieve colistin-mediated mortality to *P*. *polymyxa*. Extra addition of Mg^2+^ increases the LgCFU/mL of *P*. *polymyxa* to 3.20. On the other hand, extra addition of Ca^2+^ increases the LgCFU/mL of *P*. *polymyxa* to 4.64. Therefore, Ca^2+^ is more effective to protect colistin-subjected *P*. *polymyxa* than Mg^2+^. Furthermore, we performed a CaCl_2_ titration experiment in a range from 0 to 80 mM. As shown in Fig 2B, a positive correlation between dosage and cell survival is observed, where the protection effect of CaCl_2_ against colistin reaches saturation at about 20 mM. These results clearly demonstrate the critical protection function of divalent cations on *P*. *polymyxa* against colistin. In the following experiments, 20 mM CaCl_2_ was applied to protect *P*. *polymyxa* against colistin if necessary.

A time-dependent protection effect of CaCl_2_ on *P*. *polymyxa* against colistin was also investigated (Fig 2C). The LgCFU/mL decreases from 6.05 to 4.25 when the delay time of CaCl_2_ addition increases from 0 to 60 min. In the experimental time frame, the earlier the CaCl_2_ was added, the better the protective effect was found. Time-dependent bactericidal assay (Fig 2D) was also performed. The results show that colistin treatment causes a steady decrease of LgCFU/mL from 7.74 to 3.10 within 4 h, while extra addition of Ca^2+^ only causes the decrease of LgCFU/ml from 7.74 to 4.95 within 4 h, meaning up to around two orders of magnitude of the survival rescue. A negative control was also performed to confirm that *P*. *polymyxa* CFU would not decrease in the absence of colistin within the experimental time frame.

### Leakage of intracellular components from *P*. *polymyxa*

**Fig 3:**
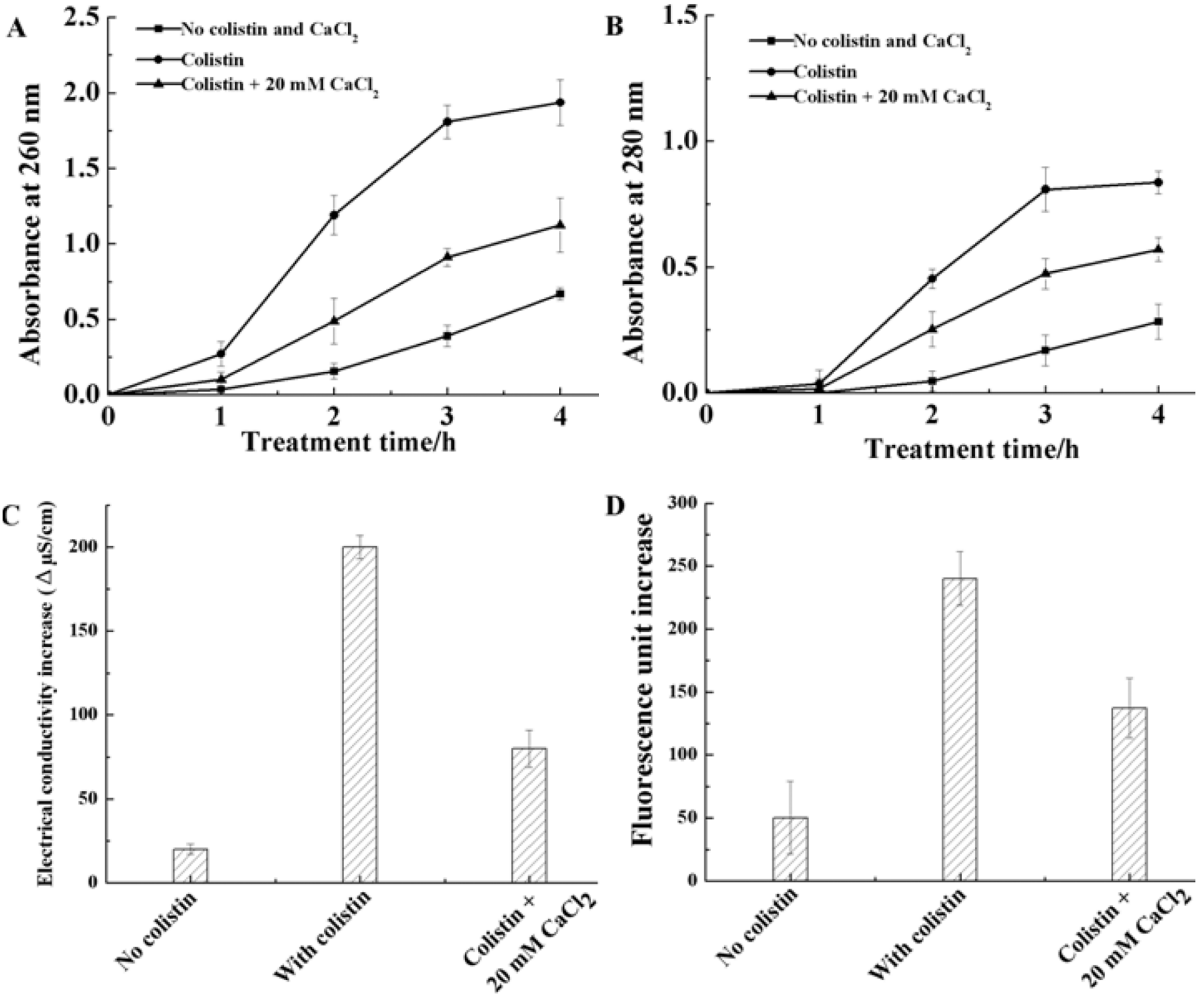
Intracellular components release due to membrane disruption caused by 1.6×10^5^ U/mL colistin and protection of CaCl_2_. (A) changes of absorbance at 260 nm over 4 h treatment. (B) changes of absorbance at 280 nm over 4 h treatment. (C) changes of electrical conductivity between at 0 h and at 2 h after treatment. (D) changes of the propidium iodide fluorescence (excitation at 535 nm and emission at 615 nm) between at 0 h and at 2 h after treatment.

Leakage of intracellular components was monitored by measuring release of macromolecules and electrolytes. The change of absorbances in 260 nm and 280 nm was used to estimate the release of intracellular macromoles, particularly nucleic acids and proteins [21]. In addition, the release of intracellular electrolytes was monitored by detecting the electrical conductivity increase [22]. In a 4h-time frame, the absorbances at 260 nm and 280 nm increase only from 0 to 0.67 (Fig 3A) and 0 to 0.28 (Fig 3B), respectively, if without any treatment. When treated with colistin, the absorbances at 260 nm and 280 nm significantly increase from 0 to 1.94 (Fig 3A) and 0 to 0.84 (Fig 3B), respectively, suggesting that the application of colistin results in higher concentration of molecules released from the cells. On the other hand, when colistin together with CaCl_2_ is applied, the absorbances at 260 nm and 280 nm moderately increase from 0 to 1.12 (Fig 3A) and 0 to 0.57 (Fig 3B), respectively, supporting that Ca^2+^ can alleviate the colistin-induced leakage of intracellular molecules. As shown in Fig 3C, similar result is obtained on electrical conductivity measurement: electrical conductivity increases the most with colistin present, the least without colistin and moderatly with colistin and CaCl_2_. These findings indicate that colistin can increase the leakage of intracellular electrolytes, while Ca^2+^ can provide certain protection from the colistin-induced damage.

PI (red fluorometric dye) staining was used for determination of cell membrane disruption [23, 24]. Once PI passes through disrupted membrane and goes into the cytoplasm, it could combine with a large amount of DNA originally existing inside the cells and emit distinctive red fluorescence signal. As shown in Fig 3D, fluorescence signal increases the most with colistin present (from 0 to 240.17), the least without colistin (from 0 to 50.27) and moderatly with colistin and CaCl_2_ together (from 0 to 137.23), demonstrating that colistin can disrupt the cell membrane and Ca^2+^ can keep the membrane integrity.

### Alteration of *P*. *polymyxa* surface morphology

**Fig 4:**
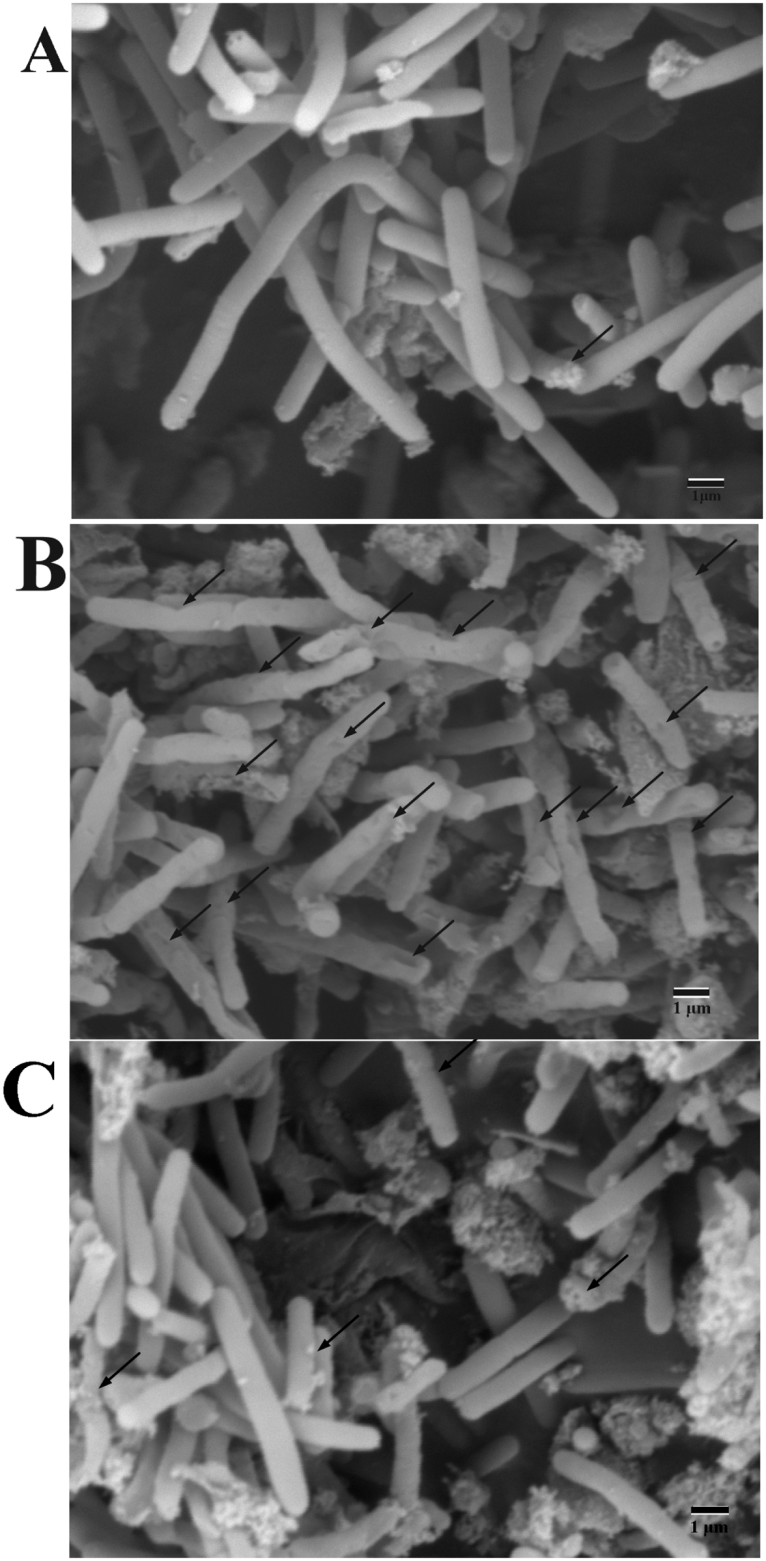
Visualization of the *P*. *polymyxa* surface morphology using SEM. (A) cells in the absence of both colistin and CaCl_2_. (B) cells treated only with 1.6×10^5^ U/mL colistin. (C) cells treated with both 1.6×10^5^ U/mL colistin and 20 mM CaCl_2_. Arrows indicate the representative irregular shapes and damaged areas on the cell surface. The scale bar is 1 μm.

The alteration of *P*. *polymyxa* surface morphology was observed under SEM [25]. As shown in Fig 4, colistin-absent *P*. *polymyxa* displays a regular smooth and plump surface. In contrast, most colistin-treated cells have irregular shapes and damaged areas on the cell surface. In the presence of both colistin and CaCl_2_, majority of *P*. *polymyxa* cells appear to be normal as the colistin-free cells, except that a few cells carry the damaged scarring. Our findings indicate that colistin can damage the cell surface integrity, but Ca^2+^ can alleviate this damage. This is in line with all our previous experiments.

## Discussion

As a cyclic lipodecapeptide, colistin carries 5 free amino groups with 5 positive charges [2]. Colistin was believed to specifically act on Gram-negative bacteria [9, 26]. Its most mentioned mechanism against Gram-negative bacteria such as *Escherichia coli*, *Pseudomonas aeruginosa*, *Klebsiella pneumoniae* and *Acinetobacter baumannii* is to kill the cells through membrane lysis by specifically targeting negatively charged LPS on Gram-negative bacteria and increasing bacterial membrane permeability. Typically, colistin at concentration of about 500 U/mL can already give bactericidal activity to many Gram-positive bacteria (data not shown). However, our study indicates that colistin at concentration only higher than 8×10^4^ U/mL can yield inhibition zones to *P*. *polymyxa*, a Gram-positive bacterium. Therefore, the concentration of colistin against Gram-positive bacteria is hundreds of times higher than that against Gram-negative bacteria. It has been found that under colistin pressure, Gram-positive bacteria will modify their teichoic acids on the cell wall through incorporation of positively charged residues, such as D-alanine, to decrease net negative charges on the cell surface for survival [16, 18]. Therefore, colistin probably targets teichoic acids on the peptidoglycan sacculi in Gram-positive bacteria, instead of LPS in Gram-negative bacteria, for electrostatic interaction. Gram-positive bacteria contain many negatively charged teichoic acids, because peptidoglycan sacculi are very thick in Gram-positive bacteria. Therefore, extraordinarily high concentration is needed for colistin to electrostatically interact with teichoic acid on the peptidoglycan sacculi in Gram-positive bacteria. Since peptidoglycan sacculi are bag-shaped molecules with relatively wide pore, this unique property enables large molecules such as proteins and peptides including colistin to diffuse through cell wall and reach the plasma membrane. Once it reaches and penetrates the plasma membrane, intracellular components will release from Gram-positive bacteria and cell will die. This explanation needs to be further investigated.

Phospholipids are the major components in the plasma membrane and play fundamental roles in forming lipid bilayers. Acidic phospholipids are present in all cell membranes, but the plasma membrane has the highest enrichment to supply lots of negative charges. Ca^2+^ ion is one of the most major intracellular cations. The membrane-proximal Ca^2+^ can directly bind to phosphate groups of acidic phospholipids, and normally function to bridge and stabilize LPS on the membrane [2, 13]. It has been found that the bactericidal activity of colistin against Gram-negative bacterium *P*. *aeruginosa* can be inhibited by Ca^2+^ ion [27]. Our data also show that Ca^2+^ ion can alleviate colistin-induced damage to Gram-positive bacterium *P*. *polymyxa*. Colistin contains 5 positive charges and enables to replace Ca^2+^ on the membrane by binding to acidic phospholipids. With the decrease of LPS stability, the plasma membrane will be disrupted and the cell will be killed by colistin. The added Ca^2+^ ions are supposed to occupy binding sites on LPS and stabilize the plasma membrane of *P*. *polymyxa*, providing protection from colistin. Interestingly, our data in Fig 2C further show that compared with the addition of Ca^2+^ immediately following colistin, the addition of Ca^2+^ with 15min delay following colistin causes about one order of magnitude further drop of *P*. *polymyxa* CFU, indicating that the colistin-induced damage to *P*. *polymyxa* is probably fast and irreversible.

Colistin is biosynthesized by *P*. *polymyxa*. Therefore, understanding of the bactericidal mechanism of colistin against its producer would not only enrich our knowledge of colistin against Gram-positive bacteria, but also provide an important guideline for optimization of fermentation condition and improvement of colistin output from *P*. *polymyxa* in the future.

## Materials and Methods

### Strain and culture condition

*P*. *polymyxa* used in this work was supplied by Zhejiang Qianjiang Biochemical Co., Ltd., China and kept frozen at -80°C in our lab at Zhejiang University of Technology, China. Unless otherwise specified, the medium for culture of *P*. *polymyxa* was followed as beef exact 10 g/L, peptone 15 g/L, glucose 10 g/L, yeast extract 2 g/L, NaCl 3 g/L and FeSO_4_·7H_2_O 0.1 g/L. To make solid medium, agar was added to a final concentration of 20 g/L. In general, the colony of *P*. *polymyxa* was first picked up using a sterilized wire loop and streaked on agar plate for incubation at 30°C for 2 d. Then, a ring of *P*. *polymyxa* was transferred to 50 mL of medium for incubation at 30°C for 18 h with a shaking at 200 rpm.

### Treatment of *P*. *polymyxa* by colistin

After cultivation, the cells were harvested by centrifugation at 4,000 g for 5 min. After washing twice with fresh medium, the cells were resuspended in fresh medium with appropriate volume to make a final cell concentration of about 10^7^ (colony forming unit or CFU/mL). Then, unless otherwise specified, colistin with final concentration of 1.6×10^5^ U/mL (supplied by Zhejiang Qianjiang Biochemical Co., Ltd., China) together with or without divalent cation (Ca^2+^ or Mg^2+^) was added into cell solution and the mixture was incubated for various times at 30°C with a shaking at 200 rpm. One unit is equal to 0.0418 μg of colistin.

### Tracking of leakage of the intracellular components from *P*. *Polymyxa* and CFU measurement

After colistin treatment with or without divalent cation, the mixture was either directly centrifuged at 4,000 g for 5 min, followed by detection of supernatant absorbances at 260 nm and 280 nm, or diluted 10 times with ultra-pure water for measurement of electrical conductivity.

To evaluate the bactericidal activity, the colistin-treated mixture was centrifuged for 5 min at 4,000 g to collect the cells. After washing twice with fresh medium, the cells were resuspended in fresh medium. Then, colony forming unit (CFU/mL) was measured on fresh agar plate after gradient dilution.

### Fluorometric assessment of the membrane permeabilization

After colistin (1.6×10^5^ U/mL) treatment with or without 20 mM Ca^2+^, the cells were harvested by centrifugation at 4,000 g for 5 min. Next, 100 μL of fluorometric dye propidium iodide (PI, 100 μg/mL) solution (Beyotime Institute of Biotechnology, Jiangsu, China) was used to resuspend the cells. After incubation at room temperature for 5 min, the cells were harvested by centrifugation at 4,000 g for 5 min and washed twice with sterilized ultra-pure water. Finally, the cells were resuspended in 1 mL of sterilized ultra-pure water and the fluorescence signal was recorded using a multimode reader (SpectraMax M2, USA) with excitation at 535 nm and emission at 615 nm.

### Visualization of the cell surface morphology by scanning electron microscopy (SEM)

The specimen was first fixed with 2.5% glutaraldehyde in phosphate buffer (pH7.0) for 4 h and washed three times with phosphate buffer (pH7.0). Then, the specimen was post-fixed with 1% hungry acid in phosphate buffer (pH7.0) for 1 h and washed three times in phosphate buffer (pH7.0). Subsequently, the specimen was gradually dehydrated by a series of ethanol (30%, 50%, 70%, 80%, 90%, 95% and 100%) for 20 min per dehydration. Next, the specimen was transferred to a mixture of alcohol and isoamyl acetate (v:v = 1:1) for a 30min-incubation and then pure isoamyl acetate for another 1h-incubation. After another dehydration in Hitachi Model HCP-2 critical point dryer with liquid CO_2_, the specimen was coated with gold-palladium and visualized with Philips Model TM-1000 SEM.
